# Fibularis tertius muscle in women & men: A surface anatomy cross-sectional study across countries

**DOI:** 10.1371/journal.pone.0215118

**Published:** 2019-04-09

**Authors:** Patricia Palomo-López, Marta Elena Losa-Iglesias, César Calvo-Lobo, David Rodríguez-Sanz, Emmanuel Navarro-Flores, Ricardo Becerro-de-Bengoa-Vallejo, Daniel López-López

**Affiliations:** 1 Centro Universitario de Plasencia, Universidad de Extremadura, Plasencia, Spain; 2 Facultad de Ciencias de la Salud, Universidad Rey Juan Carlos, Madrid, Spain; 3 Departamento de Enfermería y Fisioterapia, Instituto de Biomedicina (IBIOMED), Universidad de León, Ponferrada, León, Spain; 4 Faculty of Sport Sciences, Universidad Europea de Madrid, Villaviciosa de Odón, Madrid, Spain; 5 Facultad de Enfermería, Fisioterapia y Podologia, Universidad Complutense de Madrid, Madrid, Spain; 6 Faculty of Nursing and Podiatry, Universidad de Valencia, València, Spain; 7 Unidad de Investigación, Salud y Podología, Departamento de Ciencias de la Salud, Facultad de Enfermería y Podología, Universidade da Coruña, A Coruña, Spain; Universidade Federal Fluminense, BRAZIL

## Abstract

The fibularis tertius muscle (FTM) is a rare anatomic variation. The prevalence of this exclusively human structure, which is found in the anterior compartment of the leg, is often underestimated, and it is believed that foot and ankle conditions are more difficult to manage in patients with an FTM. The aim of this study was to assess the presence of the FTM palpation and determine whether its presence is associated with an individual’s sex, because the exact prevalence in males and females is unclear. An observational cross-sectional study was carried out. The study included 481 people (23.49% men and 76.51% women) with a mean age of 23.51±5.369 years, who were recruited from a Podiatric Medicine and Surgery Clinic (Spain). Data on routine demographic and clinical factors were recorded, and the presence or absence of the FTM was determined based on surface visual or palpated localization of the tendon (using a consistent protocol). The FTM was present in 38.25% (184/481) of the participants. Furthermore, FTM were present in 38.6% (142/481) of females and 37.2% (42/481) of males. The study revealed that the presence of the FTM varies between individuals and does not depend on an individual’s sex. Significant differences in the prevalence of the FTM between countries should be carefully evaluated rather than generalizing the results of this Spanish study to other non-Spanish populations. Larger numbers of participants should be enrolled in future studies in order to meet the statistical criteria.

## Introduction

The fibularis tertius muscle (FTM) was first described by Vesalius in 1816 [1] and was subsequently studied in detail by Henle [2] and Hyrtl [3] in the nineteenth century. It is well-documented in anatomy textbooks that the muscle forms part of the anterior compartment of the leg and crosses anterior to the ankle joint to extend up to its insertion in the anterior part of the 5^th^ and 4^th^ metatarsal bases as well as in the 5^th^ metatarsal shaft, explaining its main muscle function as an ankle evertor [4].

Also, it usually appears between the distal third and the half of the fibula from the intermuscular septum and is associated with a derivation of the extensor digitorum longus muscle, and extends into the nearby deep fascia [5–8]. The FTM is also known as the fifth tendon of this muscle [9], and its presence is inconstant among humans [10].

Anthropologically, the FTM plays an important functional and evolutionary role in the efficient terrestrial locomotion related to biomechanical role in midfoot stability [4,11] and dorsiflexion and eversion of the foot [10], and it assists in ankle dorsiflexion [12]. During human locomotion, FTM functions in combination with extensor digitorum longus and tibialis anterior muscles during the swing-phase to stabilize the foot and separate the toes from the ground. Nevertheless, FTM shows a lack of support-phase muscle activity suggesting that this muscle may not be a key muscle to support the lateral longitudinal arch [11]. From an evolutionary point of view, comparative anatomy shows a low FTM prevalence among monkeys and reaches up to 30% of prevalence in gorillas due to their almost exclusive terrestrial locomotion. The prevalence of FTM among humans as well as similar functional muscles if FTM is missing could support a key role of FTM regarding the erect bipedal posture and gait phylogenetic development [8,13].

Furthermore, this structure is innervated by the deep fibular nerve and is involved in neuromuscular control and protection against talofibular ligament injuries, which improves the efficiency and enhances the economy of human locomotion [8,14,15]. Consistently, a small nerve branch emerges from the deep fibular nerve next to the extensor digitorum longus origin, which runs parallel between extensor digitorum longus and extensor hallucis longus, and finally pierces the FTM [10]. In addition, the FTM is supplied by the anterior tibial artery similar to other muscles of the leg anterior compartment [16].

However, not only is the presence of the FTM inconsistent among humans, but its morphology can vary greatly between the right and left feet as it can have a similar bulk to the extensor digitorum longus muscle or it may be reduced to a rudimentary structure [8]. The existence of the FTM in many different populations has not yet been clearly established. It can be detected during physical evaluations and is associated with certain disorders: lateral or ankle pain [17–19], Jones fractures [20,21], tenosynovitis [22], and stress fractures [15,20], and it can be used during reconstructive surgery for ligamentous laxity [19,23] to fix a soft tissue defect in the leg or foot [24] or an ankle injury [25].

According to Vertullo et al [26], the FTM insertion site is an important factor in Jones fractures. The FTM can be used by plastic and orthopedic surgeons while performing tendoplasty, tendon transfer, or resection surgeries on the foot. Also, its muscle flap and tendon can be used for transposition to correct ankle joint laxity, and in transplantation surgeries for foot drop [27]. Therefore, the presence or absence of this muscle is important from academic and clinical points of view.

While searching the literature, we found that there were no studies regarding the anatomy of the FTM in populations in Spain. As prevalence of the FTM has not been described in Spain, the purpose of this study was firstly to determine the prevalence of the presence or absence of the FTM, and also to identify whether it is associated with an individual’s sex and whether it is more likely to be present in a Spanish population compared with populations in other countries.

## Methods

### Design and sample

A total of 500 participants were recruited from June 2016 to May 2017 for the research, which was conducted at a Podiatric Medicine and Surgery Clinic that provides treatment for diseases and disorders of the foot at University of Extremadura, Spain. The study is a cross-sectional study (S1 File) and a non-probability consecutive sampling technique was used to select the 500 participants. Nine males and ten females refused to take part in the study, while the remaining 481 individuals (113 males and 368 females) gave consent and were enrolled. The inclusion criteria were being aged ≥18 years and providing informed consent to take part in the study (taking part in an examination). The exclusion criteria were having a history of a medical condition that might affect balance, a history of trauma or lesions of the lower leg, ankle or foot, and refusal to sign the consent form or being incapable of understanding the instructions necessary to take part in the research.

### Patient and public involvement statement

The development of the research question was carried out during observation of the presence of the fibularis tertius muscle in the lower extremity from the Podiatric Medicine and Surgery Clinic of the University of Extremadura. Outcome measures were assessed by the same trained examiner after patients were informed. Patients were involved by a consecutive sampling method during recruitment. The participants were advised that the results of the study were going to be published and available for them upon request to the principal investigator.

### Sample size

The sample size was calculated with software from Unidad de Epidemiología Clínica y Bioestadística, Complexo Hospitalario Universitario de A Coruña, Universidade A Coruña (www.fisterra.com) [28]. The calculations were based on the total population living in Spain, which amounted to 42,104,557 adults on January 1, 2017. (http://www.ine.es/dyngs/INEbase/es/operacion.htm?c=Estadistica_C&cid=1254736176951&menu=ultiDatos&idp=1254735572981). It was determined that, based on a two-tailed test, a desired power of 80% (with a β level of 20%), and a precision of 5% (with an α level of 0.05) for a proportion of 50%, assuming no loss of participants, at least 384 participants must be studied. Ultimately, 481 participants were included in the study.

### Procedure

For every enrolled volunteer, the same trained examiner recorded several factors prior to the assessment using an identical protocol, comprising demographic characteristics as age, sex and BMI the inclusion criteria were able to walk independently. Next, anthropometric features were measured, i.e., height and weight with the subject barefoot and wearing short pants. The body mass index (BMI) was then calculated from the height (m) and weight (kg^2^), applying Quetelet’s equation (BMI = weight / height^2^) [29].

**Fig 1:**
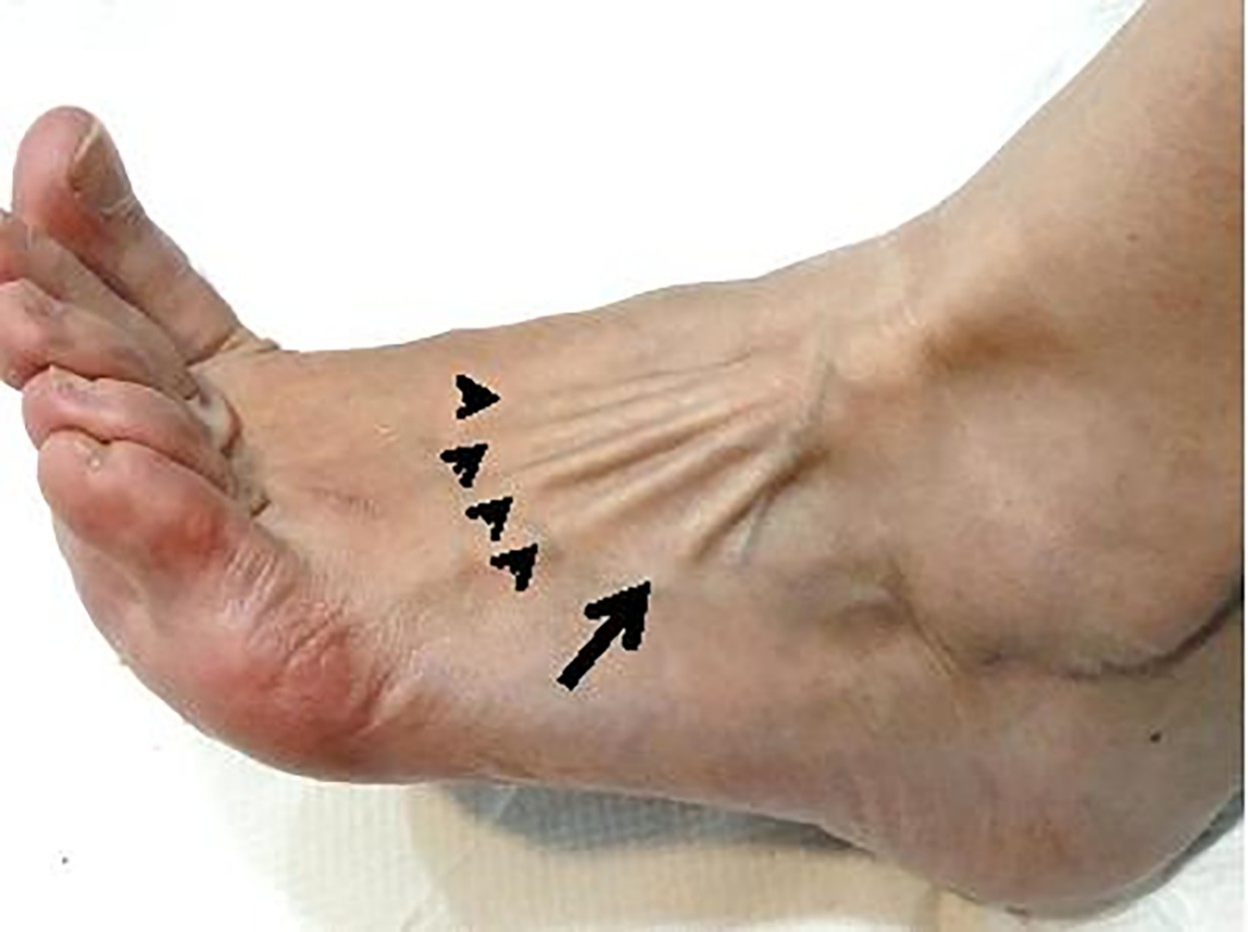
Dorsolateral view of left foot and ankle showing the tendons of the fibularis tertius (arrow) and the extensor digitorum longus tendons (arrowheads).

Finally, to determine the presence or absence of the FTM, an assessment was carried out in all subjects using the method described by Tixa [30] and Kendal [31]. Each subject sat with a relaxed posture, knee flexed at approximately 110°, and they were asked to evert the ankle and dorsiflex the toes with resistance by placing contact with the finger of the clinician along the lateral border of the foot. If the FTM tendon was visualized or felt during palpitation, this was taken to indicate the “presence” of the FTM (Fig 1). An identical protocol was used to evaluate the contralateral foot. FTM assessment by palpation of the tendon has previously been validated by comparing the results with the presence of the FTM based on magnetic resonance imaging, which indicated 100% accuracy [14].

### Ethical considerations

This research was approved by the Bioethics and Biosafety Committee at the University of Extremadura (approval code: 52/2017). All the included subjects gave their consent in written form before their inclusion in the study. Ethical standards regarding research on humans were followed, based on the Declaration of Helsinki (World Medical Association), Convention of the Council of Europe on Human Rights and Biomedicine, Universal Declaration of the United Nations Educational, Scientific and Cultural Organization (UNESCO) on the Human Genome and Human Rights, and other appropriate national or institutional organizations.

### Statistical analysis

Demographic characteristics, comprising subject age, height, weight, and BMI, were recorded, and descriptive analyses of these quantitative variables were carried out to summarize the variables using means, standard deviations (SDs), and maximum and minimum values. Categorical variables, absence or presence of the FTM, were recorded as frequencies and percentages. Data set is available as S2 File.

All the variables were examined for normality using the Kolmogorov Smirnov test, and the data were considered to be normally distributed if P > 0.05. For the variables that were normally distributed an independent t-student test was used to test for significant differences between groups. Fisher’s exact test was used to compare the qualitative variables between males and females and between countries (i.e., Spain and the countries covered in previous studies). The odds ratio (OR) for the presence/absence of the FTM, standard error, and 95% confidence interval (CI) were calculated according to Altman [32], and the associated test of significance (P-value) was calculated according to Sheskin [33].

In all of the analyses, statistical significance was established based on P < 0.05. All the analyses were performed with commercially available software (SPSS Statistics for Windows, version 19.0; IBM Corp., Armonk, NY).

## Results

A total 481 people completed all the stages of the research process, 113 of whom were male (23.49%) and 368 of whom were female (76.51%). The demographic and clinical characteristics of the sample are shown in Table 1.

**Table 1:** Demographic and clinical characteristics of the sample.

	Total Mean ± SD Range N = 481 (100%)	Male Mean ± SD Range n = 113 (23.49%)	Female Mean ± SD Range n = 368 (76.51%)	*P* value
Age (years)	23.51 ± 5.369 (18–82)	23.88 ± 5.526 (18–42)	23.40 ± 5.322 (18–82)	0.998
Weight (kg)	64.46 ± 12.137 (40–99)	76.77 ± 10.569 (49–99)	60.68 ± 9.878 (40–96)	0.001
Height (cm)	167.26 ± 8.678 (116–198)	177.40 ± 7.316 (155–198)	164.14 ± 6.385 (116–183)	0.001
BMI (kg/m^2^)	22.93 ± 3.304 (16.43–44.59)	24.35 ± 2.694 (18.70–30.10)	22.504 ± 3.356 (16.43–44.59)	0.001

Abbreviations: BMI, body mass index; SD, standard deviation. In all the analyses, *P* < .05 was considered statistically significant. The P-values are based on independent t-student test.

There was no significant difference in the presence of the FTM between male and females (P>0.05). Females were 1.062 times more likely to have FTM, with an OR of 1.062 (0.687–1.641; P = 0.786), and 0.941 times more likely to have an absence of FTM, with an OR of 0.941 (0.609–1.455; P = 0.786) compared to males, as shown in Table 2.

**Table 2:** Comparison of the presence and absence of the fibularis tertius muscle in males and females in a population in Spain.

Fibularis tertius muscle	Total Prevalence/ total (%)	Female Prevalence / total (%)	Male Prevalence / total (%)	P value*	OR (95% CI)	P value**
Presence	184/481 (38.25%)	142/481 (38,6%)	42/481 (37.2%)	0.825	1.062 (0.687–1.641)	0.786
Absence	297/481 (61.14%)	226/481 (61.4%)	71/481 (62.8%)		0.941 (0.609–1.455)	0.786

Abbreviations:

* Fisher’s Exact Test.

** OR, odds ratio. P<0.05 (with a 95% CI) was considered statistically significant. If the 95% CI of the OR contained 1, the OR was considered not statistically significant.

Regarding the FTM in the right foot, there were no significant differences in the presence or absence between males and females (P>0.05). Females were 1.062 times more likely to have the FTM in the right foot, with an OR of 1.062 (0.687–1.641; P = 0.786), and 0.941 times more likely to have an absence of the FTM in the right foot, with an OR of 0.941 (0.609–1.455; P = 0.786) compared to males, as shown in Table 3.

**Table 3:** Comparison of the presence and absence of the fibularis tertius muscle in the right and left feet in males and females in a population in Spain.

Foot	Fibularis tertius muscle	Total Prevalence/total (%)	Female Prevalence/total (%)	Male Prevalence/total (%)	P value*	OR (95% CI)	P value
Right	Presence	184/481 (38,25%)	142/481 (29,52%)	42/481 (8.73%)	0.786	1.062 (0.687-.641)	0.786
	Absence	297/481 (61,74%)	226/481 (46.98%)	71/481 (8.52%)		0.941 (0.609-.455)	0.786
Left	Presence	185/481 (38,46%)	141/481 (29,31%)	44/481 (9,14%)	0.905	0.974 (0.632-.501)	0.905
	Absence	296/481 (61,53%)	227/481 (47.19%)	69/481 (14.34%)		1.026 (0.666-.582)	1.905

Abbreviations:

* Fisher’s Exact Test.

** OR, odds ratio. P<0.05 (with a 95% CI) was considered statistically significant. If the 95% CI of the OR contained 1, the OR was considered not statistically

Regarding the FTM in the left foot, there were no significant differences in the presence or absence between male and females (P>0.05). Females were 0.941 times more likely to have the FTM in the right foot, with an OR of 0.941 (0.609–1.455; P = 0.786), and 1.026 times more likely to have an absence of the FTM at left foot, with an OR of 1.026 (0.666–1.582; P = 0.786) compared to males, as shown in Table 3. The presence or absence of the FTM in the right and left feet were similar for both sex.

A comparison of the presence of the FTM in the Spanish sample with the presence in other samples is shown in Table 4. We found that the presence of the FTM in the Spanish sample compared with a French sample was not significantly different (P = 0.0746), with the Spanish sample being 0.789 times more likely to have the FTM, with an OR of 0.789 (0.608–1.024; P = 0.0748). On the other hand, there were significant differences with all other countries (except France), including Chile, India, Belgium, Poland, Brazil, the United Kingdom, Bolivia, and Austria, as shown in Table 4, with P<0.01 (and a 95% CI for the OR that did not overlap with 1.00).

**Table 4:** Comparison of the presence and absence of the fibularis tertius muscle in Spain and other countries.

Population studied (year) N	Type of study	Presence Prevalence/ total (%)	Absence Prevalence/ total (%)	P value* Spain vs. other country	OR (95% CI) Spain vs. other country	P value
Spain (2017) N = 481. Present study.	Surface anatomy	184/481 (38.2%)	297/481 (61.7%)			
India (2015) N = 100 [40]	Cadaver dissection	87/100 (87.0%)	13/100 (13.0%)	0.0001	0.092 (0.050–0.170)	0.0001
Chile (2010) N = 168 [34]	Surface anatomy	83/168 (49.11%)	85/168 (50.5%)	0.011	0.634 (0.445–0.903)	0.011
India (2006) N = 110 [35]	Cadaver dissection	99/110 (90.0%)	11/110 (10.0%)	0.0001	0.068 (0.036–0.131)	0.0001
Belgium (2006) N = 200 [41]	Surface anatomy	163/200 (81.5%)	37/200 (18.5%)	0.00001	0.140 (0.094–0.210)	0.0001
Poland (2006) N = 193 [42]	Cadaver dissection	160/193 (83.16%)	33/193 (16.84%)	0.00001	0.127 (0.084–0.194)	0.0001
Brazil (2006) N = 32 [43]	Cadaver dissection	30/32 (93.8%)	2/32 (6.3%)	0.00001	0.041 (0.009–0.174)	0.0001
United Kingdom (2005) N = 41 [44]	Cadaver dissection	38/41 (92.7%)	3/41 (7.3%)	0.00001	0.048 (0.014–0.160)	0.0001
Bolivia (2005) N = 46 [45]	Cadaver dissection	46/46 (100.0%)	0/46 (0.0%)	0.00001	0.006 (0.0004–0.108)	0.0004
Thailand (2004) N = 247 [46]	Cadaver dissection	236/247 (95.55%)	11/247 (4.45%)	0.00001	0.028 (0.015–0.054)	0.0001
France (1991) N = 457 [47]	Cadaver dissection	201/457 (90.9%)	256/457 (9.1%)	0.0746	0.789 (0.608–1.024)	0.0748
Austria (1979) N = 169 [7]	Cadaver dissection	157/169 (92.9%)	12/169 (7.1%)	0.00001	0.0474 (0.025–0.087)	0.0001
Unknown country (Black people) (1979) N = Unknown [48]	Cadaver dissection	86/100 (86.00%)	14/100 (14.00%)	0.00001	0.100 (0.055–0.182)	0.0001
Unknown country (Jews) (1979) N = Unknown [48]	Cadaver dissection	10/100 (10.00%)	90/100 (90.00%)	0.00001	5.575 (2.828–10.991)	0.0001

## Discussion

This research focused on identifying the prevalence of the FTM in a sample from Spain compared with samples from other countries, because this issue was not previously addressed in the literature.

Ramirez et al [34] stated that the significant difference between the prevalence of the FTM reported by Witvrouw et al (81.5%) [14] and their results (49.11%) could be due to the subjects in Witvrouw et al’s sample being young athletes (with increased muscle development because of continuous physical activity), whereas Ramirez et al’s sample comprised individuals who did not undertake significant physical activity and therefore had reduced muscle development. In our study, there were participants aged from 18 to 82 years old, and we believe that the age and physical activity of the participants was not a determinant factor of the identification of the FTM because identification of the FTM by palpation is very accurate (even in obese people). However, as aponeurosis sometimes makes the FTM tendon difficult to clinically evaluate and can lead to underestimation of the prevalence, it was necessary to palpate it from the antero-lateral aspect of the ankle to its insertion.

In the present study, in the overall sample, we found that the prevalence of FTM in the right and left foot was 38.25% and 38.46%, respectively. These results are not similar to the results reported by Joshi et al [35], which were 20% and 17% in the right and left foot, respectively, nor to the results reported by Ramirez et al [34], who found a greater FTM prevalence in males compared to females, with 55.88% for the right foot and 57.35% for the left foot in males and females overall. Witvdrow et al [14] reported a prevalence of 81.6% in males and 81.4% in females. In our opinion, these results (ranging from 17% to 81.6%) are due to the different sample sizes in each study, and is necessary to calculate the minimum number of participants to include in a study to make the studies homogeneous in order to compare the results.

Regarding the ORs of the comparisons of the presence of the FTM in Spain and other countries, only the French sample indicated no significant difference. All the other countries had a significant difference. We believe that this is due to the fact that the appropriate sample sizes were not calculated in these studies, which used samples ranging from 32 participants (in the study in Brazil) to 247 participants (in the study in Thailand). These sample sizes do not meet the statistical requirements for generalizing the results to other populations or races. However, when comparing the 481 participants in our study with the 457 participants included in the study in France, the sample sizes were sufficiently large to generalize the results, and we think that this is the reason that there was no significant difference between the two countries.

Also, the significant differences may be due to non-random sampling, ethnicities sampled, and inclusion/exclusion criteria used in the various studies.

The FTM can be considered as an accessory muscle for eversion and dorsiflexion of the foot, so its absence should not significantly decrease this function, according to Witvrouw et al [14]. However, clinicians should assess its absence in athletes or older people as a possible etiologic factor that could underlie many problems involving the stability of the talocrural region.

The detection and prevalence of the FTM can be very important for foot surgeons because they can use an FTM flap transposition as an autologous tendon graft [36]. Also, the study of the FTM is relevant from academic, anatomical, physiotherapeutic, and orthopedic surgery viewpoints in terms of the treatment and/or rehabilitation of chronic instability following functional lateral ankle sprains and instability [18,37,38]. Due to its variability and possible influence on the biomechanics of the foot, further research is needed on the prevalence of the FTM and its influence [39]. Also, detailed studies of this muscle should be carried out (using surface anatomy palpation) in different population groups with normal and pathology feet and gaits in order to provide additional useful information.

## Conclusions

The present study revealed that the presence of the FTM is variable and is not influenced by an individual’s sex. In light of our results regarding significant differences in the prevalence of the FTM between countries should be carefully evaluated rather than generalizing the results of this Spanish study to other non-Spanish populations including to populations with different proportions of people from various ethnic backgrounds. Larger numbers of participants should be enrolled in future studies in order to meet the statistical criteria used in the sample size calculation in this study.

## Supporting information

S1 File Checklist for observational cross-sectional studies. (DOC)

S2 File Data set. (XLSX)

## References

[1] Vesalius A. De corporis humani fabrica libri septem. Basileae: Liber II; 1543.

[2] Henle J. Handbuch Der Systematischenanatomie Des Menschen. Muskellehre V& S, editor. Braunschweig; 1858.

[3] Hyrtl J. Lehrbuch Der Anatomie Des Menschen: Mit Rücksicht Auf Physiologische Begründung und Praktische Anwendung. Braumfille Wien; 1862.

[4] Eliot DJ, Jungers WL. Fifth metatarsal morphology does not predict presence or absence of fibularis tertius muscle in hominids. J Hum Evol. 2000;38: 333–342. 10.1006/jhev.1999.0337 10656782

[5] Moore KL, Dalley AF AA. Clinically Oriented Anatomy. Baltimore: Lippin-cott Williams and Wilkins; 2013.

[6] López PP, López DL, Sanz DR, Frutos JCP. Consideraciones en el estudio anatómico sobre el músculo peroneo tercero, peroneo anterior, tercer peroneo, peroneo tertius, fibularis tertius. Rev Int Cienc Podol. 2013; 7(1): 41–47 10.5209/rev_ricp.2013.v7.n1.41119

[7] Krammer E, Lischka M, Gruber H. Gross anatomy and evolutionary significance of the human peroneus III. Anat Embryol (Berl). 1979;155: 291–302. 572152 10.1007/BF00317642

[8] Yammine K, Erić M. The Fibularis (Peroneus) Tertius Muscle in Humans: A Meta-Analysis of Anatomical Studies with Clinical and Evolutionary Implications. Biomed Res Int. 2017;2017: 1–12. 10.1155/2017/6021707 28596965 PMC5449999

[9] Standring S. Gray’s anatomy: the anatomical basis of clinical practice.

[10] Rourke K, Dafydd H, Parkin IG. Fibularis tertius: Revisiting the anatomy. Clin Anat. Wiley Subscription Services, Inc., A Wiley Company; 2007;20: 946–949. 10.1002/ca.20500 17584875

[11] Jungers WL, Meldrum DJ, Stern JT. The functional and evolutionary significance of the human peroneus tertius muscle. J Hum Evol. 1993;25: 377–386. 10.1006/jhev.1993.1056

[12] Oyedun OS, Kanu LC, Onatola OA, Zelibe PO. Advances in Life Science and Technology. Adv Life Sci Technol. International Institute for Science, Technology and Education (IISTE); 2011;21: 69–75.

[13] Albay S, Candan B. Evaluation of fibular muscles and prevalence of accessory fibular muscles on fetal cadavers. Surg Radiol Anat. 2017;39: 1337–1341. 10.1007/s00276-017-1887-y 28608130

[14] Witvrouw E, Vanden Borre K, Willems TM, Huysmans J, Broos E, De Clercq D. The Significance of Peroneus Tertius Muscle in Ankle Injuries. Am J Sports Med. 2006;34: 1159–1163. 10.1177/0363546505286021 16493171

[15] Das S, Haji Suhaimi F, Abd Latiff A, Pa Pa Hlaing K, Abd Ghafar N, Othman F. Absence of the peroneus tertius muscle: cadaveric study with clinical considerations. Rom J Morphol Embryol. 2009;50: 509–11. 19690784

[16] Lezak B, Summers S. Anatomy, Bony Pelvis and Lower Limb, Posterior Tibial Artery [Internet]. StatPearls. 2018 Available: http://www.ncbi.nlm.nih.gov/pubmed/30725666 30725666

[17] Derrick E, Flores M, Scherer K, Bancroft L. Peroneus Tertius Tendon Tear: A Rare Cause of Lateral Ankle Pain. 2016; 10.7759/cureus.577 27226938 PMC4873314

[18] McGoldrick NP, Bergin D, Kearns SR. Peroneus Tertius Tendon Tear Causing Lateral Ankle Pain in a Child. J Foot Ankle Surg. 2017;56: 854–856. 10.1053/j.jfas.2017.02.014 28633791

[19] Sammarco GJ, Henning C. Peroneus Tertius Muscle as a Cause of Snapping and Ankle Pain. Am J Sports Med. 2007;35: 1377–1379. 10.1177/0363546506298107 17293470

[20] Vertullo CJ, Glisson RR, Nunley JA. Torsional Strains in the Proximal Fifth Metatarsal: Implications for Jones and Stress Fracture Management. Foot Ankle Int. SAGE PublicationsSage CA: Los Angeles, CA; 2004;25: 650–656. 10.1177/107110070402500910 15563388

[21] Ercikti N, Apaydin N, Kocabiyik N, Yazar F. Insertional Characteristics of the Peroneus Tertius Tendon: Revisiting the Anatomy of an Underestimated Muscle. J Foot Ankle Surg. 2016;55: 709–713. 10.1053/j.jfas.2016.01.018 26860045

[22] Cerrahisi EH, Taşer F, Shafiq Q, Toker S. Eklem Hastalıkları ve Cerrahisi Joint Diseases and Related Surgery Case Report / Olgu Sunumu. 2009;20: 165–168. 19958274

[23] Dockery GL, Toothaker J, Suppan RJ. A lateral ankle stabilization procedure utilizing the peroneus brevis and peroneus tertius tendons. J Am Podiatry Assoc. 1977;67: 891–4. 10.7547/87507315-67-12-891 925324

[24] Buarque de Gusmão LM, Brito Lima JS, Gaia Duarte FH, De Farias Souto AG, Veloso Couto BDM. Anatomical basis for the use of the fibularis tertius muscle in myocutaneous flaps. Rev Bras Cir Plást. 2013;28: 191–5.

[25] Gaulrapp H, Heimkes B. [Peroneus tertius tendon repair following old traumatic rupture of the anterior tibial tendon (casuistry)]. Unfallchirurg. 1997;100: 979–83. 9492645 10.1007/s001130050221

[26] Vertullo C, Glisson R, Nunley J. Torsional strains in the proximal fifth metatarsal: implications for Jones and stress fracture management. Foot Ankle Int. 2004;25: 650–6. 10.1177/107110070402500910 15563388

[27] Ozkan T, Tuncer S, Ozturk K, Aydin A, Ozkan S. No Title. J Reconstr Microsurg. 2009;25: 157–64. 10.1055/s-0028-1103502 19037849

[28] Pita Fernández S. Determinación del tamaño muestral. CAD ATEN PRIMARIA. 1996;3: 138–152.

[29] Garrow JS, Webster J. Quetelet’s index (W/H2) as a measure of fatness. Int J Obes. 1985;9: 147–53. Available: http://www.ncbi.nlm.nih.gov/pubmed/4030199 4030199

[30] Tixa S. Atlas de anatomía palpatoria. 2nd Editio Barcelona: Masson; 2006.

[31] Kendall FP. Músculos: pruebas funcionales, postura y dolor Madrid: Marban; 2007.

[32] Altman D. Practical statistics for medical research London: Chapman and Hall; 1991 10.1002/sim.4780101015

[33] Sheskin DJ. Handbook of Parametric and Nonparametric Statistical Procedures Chapman & Hall /CRC, editor. Boca Raton; 2004.

[34] Ramirez D, Gajardo C, Caballero P, Zavando D, Cantín M, Suazo Galdames I. Clinical evaluation of fibularis tertius muscle prevalence. Int J Morphol. 2010;28: 759–764.

[35] Joshi S, Joshi S, Athavale S. Morphology of peroneus tertius muscle. Clin Anat. 2006;19: 611–4. 10.1002/ca.20243 16317742

[36] Malagelada F, Vega J, Guelfi M, Kerkhoffs G, Karlsson J, Dalmau-Pastor M. Anatomic lectures on structures at risk prior to cadaveric courses reduce injury to the superficial peroneal nerve, the commonest complication in ankle arthroscopy. Knee Surgery, Sport Traumatol Arthrosc. 2019; 10.1007/s00167-019-05373-x 30729253

[37] Flores Santos F, Santos NR. Arthroscopic treatment of lateral ankle instability. Is there a safe zone? An anatomic study. Foot Ankle Surg. 2018; 10.1016/j.fas.2018.11.011 30563745

[38] Sanz DR, Lobo CC, López DL, Morales CR, Marín CS, Corbalán IS. Interrater Reliability in the Clinical Evaluation of Myofascial Trigger Points in Three Ankle Muscles. J Manipulative Physiol Ther. 2016;39: 623–634. 10.1016/j.jmpt.2016.09.002 27816210

[39] Yildiz S, Yalcin B. An unique variation of the peroneus tertius muscle. Surg Radiol Anat. 2012;34: 661–663. 10.1007/s00276-011-0929-0 22223362

[40] Jadhav Surekha D, Ambali Manoj P, Patil Raosaheb R, Doshi Medha A. Fibularis Tertius Muscle: Cadaveric Study in Indians. JKIMSU. 2015;4: 64–69.

[41] Witvrouw E, Vanden K, Marieke T, Huysmans J, Broos E, De Clercq D. The significance of peroneus tertius muscle in ankle injuries. Am J Sport Med. 2006;34: 1159–63. 10.1177/0363546505286021 16493171

[42] Domagata Z, Gworys B, Kreczynska B, Mogbel S. A contribution to the discussion concerning the variability of the third peroneal muscle: an anatomical analysis on the basis of foetal material. Folia Morphol (Warsz). 2006;65: 329–36. 17171612

[43] Marin L, Barbosa F, Andrade O, Bazanelli AC, Ruiz C, Pereira L, et al Estudo anatômico do músculo fibular terceiro em humanos. Arq Méd ABC. 2006;31: 23–26.

[44] Rourke K, Dafydd H, Parkin I. Fibularis tertius: revisiting the anatomy. Clin Anat. 2007;20: 946–9. 10.1002/ca.20500 17584875

[45] Larico I, Jordán L. Frecuencia del músculo peroneo tertius. Rev Inv e Info Salud. 2005;1: 29–32.

[46] Kunnika C, Jantima R, Wimon R. The presence of the peroneous tertius muscle in Thai people. SiriajHosp Gaz. 2004;56: 216–221.

[47] Bertelli J, Khoury Z. The peroneus tertius island muscle. flap Surg Radiol Anat. 1991;13: 243–4. 1754963 10.1007/BF01627997

[48] Testut L, Latarjet A. Tratado de Anatomía Humana Barcelona: Salvat; 1979.

